# Circulating Arsenic is Associated with Long-Term Risk of Graft Failure in Kidney Transplant Recipients: A Prospective Cohort Study

**DOI:** 10.3390/jcm9020417

**Published:** 2020-02-03

**Authors:** Camilo G. Sotomayor, Dion Groothof, Joppe J. Vodegel, Tomás A. Gacitúa, António W. Gomes-Neto, Maryse C. J. Osté, Robert A. Pol, Catterina Ferreccio, Stefan P. Berger, Guillermo Chong, Riemer H. J. A. Slart, Ramón Rodrigo, Gerjan J. Navis, Daan J. Touw, Stephan J. L. Bakker

**Affiliations:** 1 Department of Internal Medicine, University Medical Center Groningen, University of Groningen, 9700 RB Groningen, The Netherlands; d.groothof@umcg.nl (D.G.); j.j.vodegel@umcg.nl (J.J.V.); t.a.gacitua.guzman@umcg.nl (T.A.G.); s.p.berger@umcg.nl (S.P.B.); g.j.navis@umcg.nl (G.J.N.); s.j.l.bakker@umcg.nl (S.J.L.B.); 2 Division of Transplantation Surgery, University Medical Center Groningen, University of Groningen, 9700 RB Groningen, The Netherlands; r.pol@umcg.nl; 3 Advanced Center for Chronic Diseases, Pontificia Universidad Católica de Chile, 8330033 Santiago, Chile; catferre@gmail.com; 4 Department of Radiology, Clínica Alemana de Santiago, Universidad del Desarrollo, 7610658 Santiago, Chile; gchongm@yahoo.com; 5 Department of Nuclear and Molecular Imaging, University Medical Center Groningen, University of Groningen, 9700 RB Groningen, The Netherlands; r.h.j.a.slart@umcg.nl; 6 Institute of Biomedical Sciences, Faculty of Medicine, University of Chile, CP 8380453 Santiago, Chile; rrodrigo@med.uchile.cl; 7 Department of Pharmacy and Clinical Pharmacology, University Medical Center Groningen, University of Groningen, 9700 RB Groningen, The Netherlands; d.j.touw@umcg.nl

**Keywords:** arsenic, diet, fish consumption, oxidative stress, kidney transplantation, graft failure

## Abstract

Arsenic is toxic to many organ systems, the kidney being the most sensitive target organ. We aimed to investigate whether, in kidney transplant recipients (KTRs), the nephrotoxic exposure to arsenic could represent an overlooked hazard for graft survival. We performed a prospective cohort study of 665 KTRs with a functional graft ≥1 year, recruited in a university setting (2008‒2011), in The Netherlands. Plasma arsenic was measured by ICP-MS, and dietary intake was comprehensively assessed using a validated 177-item food-frequency questionnaire. The endpoint graft failure was defined as restart of dialysis or re-transplantation. Median arsenic concentration was 1.26 (IQR, 1.04‒2.04) µg/L. In backwards linear regression analyses we found that fish consumption (std β = 0.26; *p* < 0.001) was the major independent determinant of plasma arsenic. During 5 years of follow-up, 72 KTRs developed graft failure. In Cox proportional-hazards regression analyses, we found that arsenic was associated with increased risk of graft failure (HR 1.80; 95% CI 1.28–2.53; *p* = 0.001). This association remained materially unaltered after adjustment for donor and recipient characteristics, immunosuppressive therapy, eGFR, primary renal disease, and proteinuria. In conclusion, in KTRs, plasma arsenic is independently associated with increased risk of late graft failure.

## 1. Introduction

Arsenic is toxic to many organ systems, the kidney being the most sensitive target organ [1,2]. Free radical mediated-oxidative damage is the cornerstone of arsenic-induced pathology [3]. Arsenic induces morphological alterations of mitochondria that lead to uncontrolled formation of free radicals [4], whilst it inhibits the production of glutathione that protects cells from oxidative damage, ultimately yielding irreversible cell damage [5,6]. The kidney being a major player in removal of arsenic from the system, it is also very much exposed to arsenic and therefore susceptible to arsenic-induced toxicity [7,8,9,10].

A large variety of arsenic compounds are known, divided into the elemental metal, inorganic, and organic compounds with a large variety of toxicity [1,2,11,12]. While an extraordinary cause for arsenic intake has been described as hydroarsenicism—contamination of drinking water with arsenic in the US, Chile, and Taiwan—arsenic in food is an increasingly recognized pathway of environmental exposure. Thus, upon background regional differences, arsenic exposure substantially derives from rice consumption, as well as vegetables, fruits, and herbal tea [13,14,15,16,17,18,19]. Of note, however, seafood is thought to be a major route for arsenic intake, followed by alcohol consumption, with the latter mainly due to contaminated wine, therewith representing an evident public health threat [20,21].

Basic and clinical evidence has linked arsenic exposure to nephrotoxicity, tubular necrosis, diffuse interstitial fibrosis, decline of kidney function, incident chronic kidney disease, and progress of native chronic kidney disease, among several other conditions such as hypercalciuria, albuminuria, and nephrocalcinosis [22,23,24,25,26,27,28,29]. Kidney transplant recipients (KTRs) are particularly vulnerability to the harmful effects of nephrotoxic agents. However, no study has been devoted to evaluating whether arsenic may be an otherwise overlooked modifiable risk factor in the post-kidney transplantation setting. The current study, therefore, aimed to identify independent environmental and system determinants of plasma arsenic levels and to evaluate the potential association of plasma arsenic levels with long-term risk of graft failure in a large cohort of well-characterized KTRs.

## 2. Methods

### 2.1. Design and Study Population

In this prospective cohort study, outpatient adult KTRs with a functioning graft ≥1 year, no alcohol or drug addiction, and without known systemic illnesses (i.e., malignancies, opportunistic infections) were invited to participate. The recruitment of patients took place at the University Medical Center Groningen between November 2008 and March 2011. In total, 817 KTRs were invited for the study, of whom 707 (87%) provided written informed consent to participate. All patients with missing plasma arsenic levels were excluded, resulting in 665 KTRs eligible for statistical analyses. Multiple imputations (*n* = 5) were used to account for missingness of data among variables other than data on circulating arsenic. The present study was approved by the Institutional Review Board (METc 2008/186) and was conducted in accordance with the Declaration of Helsinki.

The primary outcome of this study was death-censored graft failure, defined as end-stage kidney disease requiring dialysis or re-transplantation. The continuous surveillance system of the outpatient clinic of our university hospital, in which patients visit the outpatient clinic with declining frequency in accordance with the American Transplantation Society Guidelines, ensured updated information on patient status [30]. General practitioners or referring nephrologists were contacted in case the status of a patient was unknown. Endpoints were recorded until September 2015. No patients were lost to follow-up.

All KTRs were transplanted at the University Medical Center Groningen following the establishment of standard antihypertensive and immunosuppressive therapies. Relevant characteristics including recipient age, gender, cardiovascular history, and transplant-related information were extracted from patient records. Dietary intake, clinical parameters, and laboratory measurements were extensively assessed at baseline. 

### 2.2. Assessment of Dietary Intake

Dietary intake was assessed using a validated semi-quantitative food frequency questionnaire (FFQ) developed and updated at Wageningen University [31]. The questionnaire consisted of 177 food items to record intake during the last month, taking seasonal variations into account. For each item, the frequency was expressed in times per day, week, or month. The number of servings was recorded in natural units (e.g., slice of bread or apple) or household measures (e.g., cup or spoon). The FFQ was self-administered and then checked by a trained researcher on the day of visit to the outpatient clinic. Inconsistent answers were verified with the patients. The results of the FFQ were converted into total energy and nutrient intake per day by using the Dutch Food Composition Table of 2006 [32].

### 2.3. Clinical Parameters and Definitions

All measurements were performed during a morning visit to the outpatient clinic. Blood pressure was determined with a semi-automatic device (Dinamap 1846, Critikon, Tampa, FL, USA), measuring every minute for 15 min. The last three measurements were averaged, following a strict protocol as described previously [33]. Body mass index (BMI) was calculated as weight in kilograms divided by height in meters squared (kg/m^2^), and body surface area (BSA) was estimated in meters squared (m^2^) by using the universally adopted formula of DuBois and DuBois [34]. Diabetes was defined as use of antidiabetic medication, fasting plasma glucose ≥ 7.0 mmol/L, and/or HbA_1C_ higher than 6.5% [35]. Kidney function was assessed by means of estimated glomerular filtration rate (eGFR) according to the Chronic Kidney Disease Epidemiology Collaboration equation [36].

### 2.4. Laboratory Methods and Arsenic Measurement

Blood was drawn after a fasting period of 8–12 h, which included no medication intake. Serum high-sensitivity C-reactive protein (hs-CRP), HbA_1C_, triglycerides, low-density lipoprotein (LDL) cholesterol, high-density lipoprotein (HDL) cholesterol, and total cholesterol were measured using routine laboratory methods. Serum creatinine was determined using a modified version of the Jaffé method (MEGA AU 510, Merck Diagnostica, Darmstadt, Germany). Serum cystatin C was determined using Gentian Cystatin C Immunoassay (Gentian AS, Moss, Norway) on a modular analyzer (Roche Diagnostics, Mannheim, Germany). Class I and class II human leukocyte antigens (HLA) antibodies were assessed by ELISA (LATM20×5, One Lambda, Canoga Park, CA, USA) as described elsewhere [37]. According to a strict protocol, all participants were instructed to collect a 24 h urine sample the day before to their visit to the outpatient clinic. Total urinary protein concentration was determined using the Biuret reaction (MEGA AU 150, Merck Diagnostica, Darmstadt, Germany). 

Arsenic plasma concentrations were assessed from EDTA plasma samples that were stored frozen at −80 °C. Arsenic plasma concentrations were determined using inductively coupled plasma mass spectrometry (ICP-MS, Varian 820-MS; Varian, Palo Alto, CA, USA) with a modified method for the measurement of low concentrations of heavy metals in plasma using a standard addition method. Standards were made by addition to blanc plasma known amounts of arsenic to obtain added concentrations of 0.500, 1.00, 2.00, 3.00, 4.00, and 5.00 μg/L. Control samples were made by spiking blanc plasma with known amounts of arsenic to obtain added concentrations of, respectively, 0.75 (low), 2.5 (medium), and 4.5 μg/L (high). Sample preparation consisted of diluting 100 µL sample with 1.0 mL dilution reagent. The dilution reagent contained 0.005% Triton X100, 0.005% EDTA, and 0.1 mg/L Yttrium as internal standard. Characteristics of this method are summarized in Table 1.

### 2.5. Follow-Up of Plasma Arsenic Levels in a Sample Population of the TransplantLines Cohort and Biobank Study

Additionally, to investigate plasma arsenic levels over time, we requested follow-up plasma samples (3 months, 6 months, 1 year, and 2 years post-kidney transplantation) from 46 consecutive KTRs enrolled between February 2016 and May 2017 in the ongoing TransplantLines Prospective Cohort and Biobank Study [38]. Arsenic plasma concentrations were determined using inductively coupled plasma mass spectrometry (ICP-MS, Varian 820-MS; Varian, Palo Alto, CA, USA) with a modified method for the measurement of low concentrations of heavy metals in plasma using a standard addition method, as described hereby in the preceding section.

### 2.6. Statistical Analyses

Data analyses were performed using SPSS version 23.0 software (SPSS, Inc., Chicago, IL, USA) and R version 3.2.3 (R Foundation for Statistical Computing, Vienna, Austria). Continuous variables were summarized using mean (SD) for normally distributed data, whereas skewed distributed variables are given as median (IQR). Categorical variables were summarized as numbers (percentage). In all analyses, a two-sided *p* < 0.05 was considered significant. Linear regression analyses were performed to evaluate the association of baseline characteristics with arsenic concentrations, adjusted for (i) age and sex, and additionally (ii) eGFR. The assumption of homoscedasticity and normality of residual variance were verified, and a natural log-transformation was applied when appropriate. Std. β coefficients represent the difference (in SD) in arsenic per 1-SD increment in continuous characteristics or for categorical characteristics the difference (in SD) in arsenic compared to the implied reference group. In order to study, in an integrated manner, which baseline characteristics were independently associated with and were determinants of plasma arsenic, we performed forward selection of baseline characteristics according to preceding multivariable linear regression analyses (*p* for inclusion < 0.2), followed by stepwise backwards multivariable linear regression analyses (*p* for exclusion 0.05). Finally, we also performed a stepwise backwards multivariable linear regression with exclusion of eGFR in the initial model in order to isolate environmental determinants of plasma arsenic levels. 

The prospective association of plasma arsenic with risk of graft failure during follow-up was examined incorporating time to event and accounting for death-censoring, by means of univariable and multivariable Cox proportional-hazards regression analyses with time-dependent covariates to calculate hazard ratios (HR) and 95% confidence intervals (CI). Schoenfeld residuals were calculated to assess whether proportionality assumptions were satisfied. Associations are shown with plasma arsenic as a continuous variable and according to tertiles of the plasma arsenic distribution. Following univariable analyses (model 1), we first performed multivariable adjustment for the most important environmental determinants of arsenic levels according to the results of our backwards linear regression analyses (model 2). To avoid overfitting, further models were performed with additive adjustments to model 2, defined as the primary multivariable model [39]. Thus, we performed additional adjustments for intake of fruits, vegetables, potato, rice, bread, and total energy intake (model 3); transplant characteristics (donor and recipient age, donor type, HLA mismatches, circulating anti-HLA class I antibodies, circulating anti-HLA class II antibodies, transplant vintage, and immunosuppressive therapy; model 4); risk factors of graft failure (eGFR, hs-CRP, systolic blood pressure, total cholesterol, and triglycerides concentration; model 5); and primary renal disease and proteinuria in model 6.

The intra-individual coefficient of variation (CV) for plasma arsenic levels in KTRs of the TransplantLines Cohort and Biobank Study was calculated using the formula CV = (SD/mean) × 100, in which SD is the standard deviation and mean is the mean value for plasma arsenic concentrations as measured in follow-up samples taken at 3 months, 6 months, 1 year, and 2 years post transplantation. Next, box plots were used to illustrate medians (interquartile range) of plasma arsenic levels during follow-up visits. Finally, significance of potential change during follow-up visits was tested using the Kruskal Wallis test.

## 3. Results

### 3.1. Baseline Characteristics and Cross-Sectional Analyses

Mean (SD) age of the 665 KTRs was 53 (13) years, of whom 383 (58%) were male. Median (IQR) plasma arsenic concentration was 1.26 (1.04–−2.04) µg/L. The baseline characteristics of the study participants along with the results of age- and sex- as well as eGFR-adjusted linear regression analyses are shown in Table 2. In stepwise backward multivariable linear regression analysis, fish consumption (β = 0.26; *p* < 0.001), eGFR (β = −0.11; *p* = 0.02), and proteinuria (std β = 0.18; *p* < 0.001) were identified as independent determinants of plasma arsenic concentrations (Table 2). If analyses were performed with eGFR excluded from the initial model, fish consumption (β = 0.27; *p* < 0.001) was identified as the only independent determinant of arsenic (Table 2). 

### 3.2. Prospective Analyses

**Figure 1:**
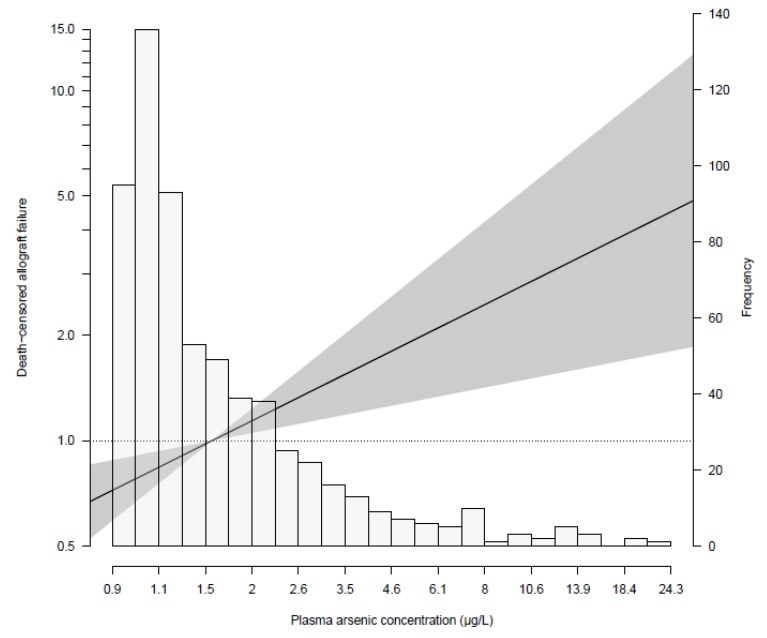
Association between plasma arsenic concentration and risk of death-censored graft failure using Cox regression analyses with mean concentration of plasma arsenic as reference, adjusted for age, sex, fish intake, and alcohol consumption, and in relation to the histogram of plasma arsenic distribution.

During a follow-up of 5 years, 72 (11%) patients developed graft failure. Chronic allograft dysfunction was the major cause of graft failure accountable for 50 (69%) of all graft failures. Other causes for graft failure included return of primary kidney disease (11%), infection (4%), acute rejection (4%), BK nephropathy (4%), vascular complications (3%), and others (4%). From low to high tertiles of the plasma arsenic distribution, 18, 25, and 29 patients developed graft failure, respectively. Prospective analyses of the association of plasma arsenic with death-censored graft failure are shown in Table 3. Multivariable-adjusted Cox proportional hazards models showed that plasma arsenic was directly associated with graft failure (HR 1.80; 95% CI 1.28–2.53, *p* = 0.001), independent of major environmental determinants of arsenic concentration, i.e., alcohol and fish consumption. In analyses with further adjustment for potential confounders, the association remained materially unchanged (Table 3). We did not find signs of a non-linear association between plasma arsenic levels and risk of death-censored graft failure (Supplementary Materials Table S1). Figure 1 illustrates the association between plasma arsenic concentration and risk of death-censored graft failure using Cox regression analyses with mean concentration of plasma arsenic as reference, adjusted for age, sex, fish intake and alcohol consumption, and in relation to the histogram of plasma arsenic distribution.

### 3.3. Follow-up of Plasma Arsenic Levels in a Sample Population of the TransplantLines Cohort and Biobank Study

In Supplementary Materials Figure S1 we show box plots with medians (IQR) of plasma arsenic concentration of 46 KTRs (mean age 52 ± 14 years-old, eGFR 43 ± 28 mL/min/1.72 m^2^) from the TransplantLines Prospective Cohort and Biobank Study, at different follow-up visits post-kidney transplantation. Median (interquartile range) plasma arsenic concentrations were 1.61 (1.51–1.99), 1.64 (1.52–2.05), 1.64 (1.43–1.94), and 1.59 (1.46–2.26) µg/L at 3 months, 6 months, 1 year, and 2 years post-kidney transplantation, respectively. Median (interquartile range) intra-individual coefficient of variation was 12.2% (6.7–28.7%), and we did not find signs of a significant change in plasma arsenic levels over time (*p* = 0.64).

## 4. Discussion

In these analyses of 665 well-characterized individuals from a Dutch cohort of KTRs, we identified fish consumption as the major environmental determinant of plasma arsenic levels. Prospective analyses showed that higher plasma arsenic levels are associated with increased long-term risk of graft failure, independent of donor and recipient characteristics, immunosuppressive therapy, eGFR, and proteinuria. These data pose arsenic as a potentially modifiable risk factor for late graft failure in KTRs, emphasizing the need for specific recommendations regarding arsenic exposure, as well as patient monitoring and management of arsenic-induced kidney injury, particularly in populations highly susceptible to nephrotoxic agents such as KTRs.

Being the major organ involved in arsenic clearance, the kidney is highly susceptible and the most sensitive target organ to arsenic exposure [1,2,9,10]. Arsenic-induced oxidative stress has been suggested to be the cornerstone of pathological mechanisms leading to kidney injury and development of chronic kidney disease [3,40]. On the one hand, decreased antioxidant capacity has been shown in individuals exposed to arsenic [41], wherein depletion of glutathione has been consistently described [5,42,43]. Of note, by protecting cells from oxidative damage, inhibition of glutathione production and subsequent glutathione depletion ultimately reverberates into increased vulnerability of cells to arsenic damage. On the other hand, it has been shown that arsenic induces morphological alterations of mitochondrial integrity that lead to uncontrolled free radical formation [4], which further feeds the circle of oxidative challenge and tissue injury. Indeed, basic and clinical evidence has linked arsenic exposure to nephrotoxicity, tubular necrosis, diffuse interstitial fibrosis, decline of kidney function, incident chronic kidney disease, and progress of native chronic kidney disease, amongst other conditions such as hypercalciuria, albuminuria, and nephrocalcinosis [22,23,24,25,26,27,28,29]. Subsequently, diminished kidney clearance of arsenic and enhanced production of reactive oxygen species longitudinally contribute to perpetuate tissue insult and progression of chronic kidney disease [22,23]. Previous studies have also shown an association between arsenic and hypertension and type 2 diabetes mellitus, both suggesting additional mechanisms for secondary kidney damage [44,45]. Ecological studies from the United States, Chile, and Taiwan have shown that arsenic exposure is associated with increased mortality from kidney disease [13,14,15,22,26,28,46,47,48,49]. KTRs are particularly vulnerable to harmful effects of nephrotoxic agents. End-stage kidney disease and maintenance immunosuppressive therapy are constant sources of oxidative challenge for the graft tissue, which shortens the capacity of oxidative stress defenses against additional environmental hazards. To our knowledge, the current study is the first to provide evidence of an independent prospective association between circulating arsenic levels and risk of late kidney graft failure.

Further supportive evidence for the key role of oxidative stress in arsenic-induced pathogenic mechanisms—and suggestive of potential management alternatives—was provided by the observation that co-administration of ascorbic acid and α-tocopherol to arsenic-exposed rats led to a reduction in the levels of lipid peroxidation, protein carbonyls, and hydrogen peroxide along with increased levels of reduced glutathione, ascorbic acid, and α-tocopherol. Investigation aimed to evaluating whether ascorbic acid and α-tocopherol supplementation may improve arsenic-induced altered microsomal functions in the kidney is warranted [50].

An increasing body of evidence supports that the kidney is a primary site of arsenic uptake and accumulation. Recently, X-ray fluorescence spectrometry allowed detection of arsenic accumulation, specifically at level of the kidney cortex [51]. X-ray fluorescence spectrometry may provide comprehensive information of bioaccumulation for biomedical and toxicological research by allowing direct measurement of the distribution of arsenic at tissue, cellular, and subcellular level. Next, X-ray absorption spectroscopy has been shown to allow in vivo assessment of whole-body distribution, which is key information for the development of chelation therapies [52]. Future studies using these analytical methods may provide essential research data to understand the sequence of specific mechanisms of nephrotoxicity and deepen the understanding of the association between long-term arsenic exposure and kidney damage [51].

The current study is etiological in nature, which needs to be separated from prediction research [53]. Whereas the latter is a distinct field of epidemiologic research aimed at predicting the risk of an outcome according to a model of statistically significant predictors, which not necessarily represents causal associations, etiological studies aim to understand a certain pathway of a disease in an attempt to prevent its onset or progression [53]. Taken together, our findings and the aforementioned studies may support an etiological role of arsenic in pathways of disease that contribute to increased risk of death-censored graft failure.

Data on the average diet-derived arsenic exposure in The Netherlands are scarce. One study reported an estimated median (range) exposure of 37.8 (20.6–70.1) μg/day [54]. This was corroborated by a more recent study of Hoogenboom et al. stating that the average diet-derived arsenic exposure is <50 μg/day. In agreement with our findings, higher intake of arsenic most frequently originates from higher fish consumption [55]. A monitoring program from the Dutch Agriculture Advisory Committee (LAC), conducted in the 1980s, demonstrated that levels of arsenic in fish landed in The Netherlands varied between 0.8 and 6.8 mg/kg wet weight, showing a slight decreasing trend over time. Likewise, the arsenic levels in shrimps decreased from 4.3 to 1.3 mg/kg wet weight during that period (LAC program, 1991, in reference [41]). However, more recent data regarding arsenic-contaminated fish landed in The Netherlands are lacking and needed to evaluate strategies aiming to reduce the dietary consumption of arsenic by the population. Next, although in The Netherlands, naturally occurring arsenic concentrations in drinking water are usually below the concentrations required by the European drinking water standard (<10 µg/L in all countries, except Denmark, where it is <5 µg/L), health risks cannot be excluded at this level, and it has been recommended to optimize water supply to arsenic levels <1 µg/L [56,57].

The current study was performed in a large cohort of extensively phenotyped KTRs, allowing us to control our main findings for several potential confounders, including donor and recipient characteristics, immunosuppressive therapy, proteinuria, and eGFR. Moreover, patients were monitored for an extensive period and patient status was updated without losses to follow-up, allowing the study of the long-term association of arsenic with graft failure. Despite considerable improvement of short-term graft survival during last decades, improvement of long-term outcomes continues to lag behind, emphasizing that future advances in the field of kidney transplantation are expected from the amelioration of long-term graft attrition [58]. Systematic description of modifiable risk factors is key to promote preventive strategies particularly addressed for this population of solid organ patients. 

Our study derived from a single university center from the northern part of The Netherlands, which calls for prudence to extrapolate our results to different populations regarding potential environmental arsenic contamination and exposure. Additionally, the observational design of the current study does not allow hard conclusions on causality, nor could the potentiality of reversed causation or residual confounding be eliminated, despite the substantial number of potential confounders for which we adjusted. Furthermore, the technique used in the current study does not allow different species of arsenic to be distinguished, while arsenic species have major varieties in toxicity [1,2,3,4,11,12]. Elemental arsenic is nontoxic as the metal is insoluble in bodily fluids, and inorganic species of arsenic, e.g., arsenite and arsenate, are especially toxic to humans. Organic species vary in toxicity; the most common species, monomethylarsonic acid and dimethylarsinic acid, are less toxic compared to inorganic species, and arsenobetaine and arsenosugars have a very low toxicity [1,5,9,11,59,60,61]. Further studies utilizing techniques with the ability to distinguish between the different species of arsenic, e.g., high-performance liquid chromatography–inductively coupled plasma-mass spectrometry, could provide more information on the impact of the different species on graft failure in KTRs. A further limitation is that adjustment for immunological factors as potential confounders of the association was limited to adjustment for HLA matching, circulating anti-HLA class I antibodies, and circulating anti-HLA class II antibodies, since we had no data on donor-specific anti-HLA antibodies and biopsy findings. Finally, it should be acknowledged that graft failure can be the consequence of multiple, heterogenous causes. Unfortunately, in our study the numbers of cause-specific cases of death-censored graft failure was too small to allow for meaningful separate analyses [62]. Larger studies are warranted to comprehensively evaluate the association of plasma arsenic with different causes of death-censored graft failure. It should be noticed, however, that this study is the first to indicate a prospective association of arsenic with the hard endpoint graft failure, thus holding a plea for future studies which to only investigate arsenic plasma concentrations, but also take into account concentrations of arsenic in drinking water, and not only in KTRs to investigate associations with death-censored graft failure, but also in other populations, such as patients with diabetes and the general population. 

## 5. Conclusions

In conclusion, the current study shows for the first time that circulating arsenic levels are independently associated with higher risk of late kidney graft failure, emphasizing the need for specific recommendations regarding arsenic exposure, as well as patient monitoring and management of chronic arsenic-induced kidney damage. Our findings point towards arsenic as an otherwise overlooked modifiable risk factor for adverse long-term kidney outcomes, especially in populations of vulnerability to oxidative stress challenge, *e.g.*, KTRs. Further studies are warranted to confirm our results and investigate the longitudinal association between arsenic exposure and graft failure in KTRs from populations with different dietary and environmental exposure. 

## Figures and Tables

**Table 1:** Bias and precision of arsenic measurements.

Cadmium Concentration	*n*	µg/L	Bias (%)	Inter-Assay Coefficient	
				SD (µg/L)	CV (%)
Low	36	0.75	−13	0.26	40
Medium	36	2.5	−9.2	0.38	17
High	37	4.5	−6	0.48	11

*n*, number of control samples; SD, standard differentiation; CV, coefficient of variation.

**Table 2:** Baseline characteristics of 665 kidney transplant recipients (KTRs) and their association with plasma arsenic.

Baseline Characteristics		Overall KTRs *n* = 665	^†^ Plasma Arsenic (ln), µg/L	^‡^ Plasma Arsenic (ln), µg/L	Backwards Linear Regression	^§^ Backwards Linear Regression
			Std. β	Std. β	Std. β	Std. β
Plasma arsenic, µg/L, median (IQR)		1.26 (1.04–2.04)	−	−	−	−
**Demographics and body composition**						
	Age, years, mean (SD)	53 (13)	−	−		
	Sex (male), *n* (%)	383 (58)	−	−		
	Diabetes mellitus, *n* (%)	160 (24)	−0.07 *	−0.07 *	~	~
	Body surface area, m^2^, mean (SD)	1.94 (0.22)	−0.02	−0.05		
	Body mass index, kg/m^2^, median (IQR)	26.0 (23.3–29.4)	−0.003	−0.02		
	Waist circumference, cm, mean (SD)	99 (14)	0.003	−0.02		
**Cardiovascular history and lifestyle**						
	History of cardiovascular disease, *n* (%)	325 (49)	−0.01	−0.01		
	Heart rate, beats per minute, mean (SD)	69 (12)	0.01	0.02		
	Systolic blood pressure, mmHg, mean (SD)	136 (17)	−0.04	−0.06 *	~	~
	Use of antihypertensives, *n* (%)	586 (88)	0.001	−0.04		
	Current or former smoker, *n* (%)	382 (57)	0.04	0.03		
	Alcohol consumption > 10 g/d, *n* (%)	169 (25)	0.14 ***	0.14 ***	~	
**Dietary intake**						
	Bread, g/day, mean (SD)	133 (59)	−0.09 **	−0.08 *	~	~
	Vegetables, g/day, median (IQR)	90 (50–118)	−0.03	−0.03		
	Fruit, g/day, median (IQR)	123 (61–232)	−0.04	−0.04	~	~
	Potato, g/day, median (IQR)	119 (72–161)	−0.11 ***	−0.11 **	~	~
	Rice, g/day, median (IQR)	15 (4–32)	0.07 *	0.06 *	~	~
	Fish, g/day, median (IQR)	11 (4–21)	0.32 ***	0.31 ***	0.26 ***	0.27 ***
	Coffee, mg/day, median (IQR)	500 (250–625)	−0.001	0.01		
	Tea, mg/day, median (IQR)	250 (54–375)	0.03	0.01 *	~	~
**Laboratory measurements**						
	Albumin, g/L, mean (SD)	43 (3)	−0.05	−0.03		
	Calcium, mmol/L, mean (SD)	2.40 (0.15)	−0.06 *	−0.04		
	Phosphate, mmol/L, mean (SD)	0.97 (0.21)	0.09 **	0.03		
	eGFR, mL/min/1.73 m^2^, mean (SD)	53 (20)	−0.18 ***	−	−0.11 **	−
	Proteinuria, *n* (%)	150 (23)	0.12 ***	0.09 **	0.18 ***	
	Alkaline phosphatase, U/L, median (IQR)	67 (54–84)	0.02	0.02		
	ASAT, U/L, median (IQR)	22 (18–27)	0.06 *	0.07 *	~	~
	ALAT, U/L, median (IQR)	19 (14–25)	0.01	0.04		
	Gamma-GT, U/L, median (IQR)	26 (18–41)	0.05 *	0.05		
**Lipids**						
	Total cholesterol, mmol/L, mean (SD)	5.1 (1.1)	0.03	0.02		
	HDL cholesterol, mmol/L, median (IQR)	1.3 (1.1–1.6)	0.04	0.08 *	~	~
	LDL cholesterol, mmol/L, mean (SD)	3.0 (0.9)	0.02	0.01		
	Triglycerides, mmol/L, median (IQR)	1.7 (1.2–2.3)	−0.01	−0.04		
**Inflammation and oxidative stress**						
	Leukocyte count, per 10^9^/L, mean (SD)	8.1 (2.6)	0.01	0.01		
	hs-CRP, mg/L, median (IQR)	1.6 (0.7–4.5)	−0.01	−0.02		
	Malondialdehyde, µmol/L, median (IQR)	2.5 (1.9–3.7)	−0.02	−0.01		
**Primary kidney disease and kidney transplantation**						
	*Primary kidney disease*					
	Glomerulosclerosis, *n* (%)	190 (29)	0.02	0.01		
	Glomerulonephritis, *n* (%)	51 (8)	0.01	−0.01		
	Tubulointerstitial nephritis, *n* (%)	76 (11)	0.05	0.06		
	Polycystic kidney disease, *n* (%)	136 (21)	−0.09	−0.07		
	Kidney hypo/dysplasia, *n* (%)	29 (4)	0.02	0.02		
	Renovascular disease, *n* (%)	38 (6)	−0.05	−0.04		
	Diabetes, *n* (%)	32 (5)	0.04	0.04		
	Other/miscellaneous, *n* (%)	113 (17)	0.02	0.02		
	Donor type, living *n* (%)	229 (34)	−0.05	−0.04		
	Donor age, years, median (IQR)	46 (31–54)	−0.01	−0.06 *	~	~
	Transplant vintage, years, median (IQR)	5.5 (2.0–11.9)	−0.03	−0.01		
	*Immunosuppressive therapy*					
	Prednisolone dose, grams, median (IQR)	10.0 (7.5–10.0)	0.01	0.02		
	Use of calcineurin inhibitor, *n* (%)	381 (57)	0.05	0.003		
	Use of proliferation inhibitor, *n* (%)	553 (83)	−0.001	0.02		
	Acute rejection treatment, *n* (%)	176 (26)	0.04	0.03		

* *p* < 0.2; ** *p* < 0.05; *** *p* < 0.01. ^†^ Linear regression analysis; adjusted for age, sex, ^‡^ and eGFR. Std β coefficients represent the difference (in SD) in arsenic per SD increment in continuous characteristics or for categorical characteristics the difference (in SD) in arsenic compared to the implied reference group. For inclusion and exclusion in stepwise backwards linear regression analyses *p* values were set at 0.2 and 0.05, respectively. ^§^ eGFR was removed from the initial model. ~ Excluded from the final models. ALAT, alanine aminotransferase; ASAT, aspartate aminotransferase; eGFR, estimated glomerular filtration rate; HDL, high-density lipoprotein; HLA, human leukocyte antigens; hs-CRP, high-sensitivity C-reactive protein; LDL, low-density lipoprotein.

**Table 3:** Prospective analyses of the association of plasma arsenic with death-censored graft failure in 665 kidney transplant recipients.

	Plasma Arsenic				
	Tertile 1	Tertile 2	Tertile 3	Continuous (ln)	
	Ref.	HR (95% CI)	HR (95% CI)	HR (95% CI)	*p*
*n*_events_	18	25	29	72	
Model 1	1.00	1.41 (0.77–2.59)	1.69 (0.94–3.04)	1.47 (1.08–2.01)	0.02
Model 2	1.00	1.58 (0.86–2.92)	2.12 (1.14–3.95)	1.80 (1.28–2.53)	0.001
Model 3	1.00	1.55 (0.84–2.87)	2.05 (1.10–3.82)	1.74 (1.24–2.45)	0.001
Model 4	1.00	1.40 (0.75–2.61)	2.00 (1.06–3.77)	1.90 (1.32–2.73)	0.001
Model 5	1.00	1.32 (0.71–2.45)	1.76 (0.93–3.32)	1.56 (1.10–2.23)	0.01
Model 6	1.00	1.29 (0.70–2.40)	1.84 (0.99–3.42)	1.53 (1.09–2.14)	0.01

Cox proportional-hazards regression analyses were performed to assess the association of plasma arsenic with risk of death-censored graft failure (number of events = 72). Associations are shown with plasma arsenic concentration as a continuous variable and according to tertiles of the plasma arsenic distribution (tertile 1: ≤1.1 µg/L; tertile 2: 1.1‒1.67 µg/L; tertile 3: ≥1.67 µg/L). Model 1 is univariable. Multivariable model 2 was adjusted for fish intake and alcohol consumption. Subsequently, additive adjustment was performed for intake of fruits, vegetables, potato, rice, bread, and total energy intake (model 3); donor and recipient age, donor type, human leukocyte antigen mismatches (HLA), circulating anti-HLA class I antibodies, circulating anti-HLA class II antibodies, transplant vintage, and immunosuppressive therapy (model 4); eGFR, high-sensitivity C-reactive protein, systolic blood pressure, total cholesterol, and triglyceride concentration (model 5); primary kidney disease and proteinuria (model 6).

## Supplementary Materials

The following are available online at https://www.mdpi.com/2077-0383/9/2/417/s1, Figure S1: Plasma arsenic concentration of 46 kidney transplant recipients from the TransplantLines Prospective Cohort and Biobank Study [38], at different follow-up visits after transplantation, Table S1: Verification of linearity of the association between plasma arsenic and risk of death-censored graft failure.
