# Supplementary material for: Circulating Arsenic is Associated with Long-Term Risk of Graft Failure in Kidney Transplant Recipients: A Prospective Cohort Study
Source: J Clin Med. 2020 Feb 3;9(2):417. doi: 10.3390/jcm9020417 (PMC7073559; doi:10.3390/jcm9020417)
Supplement: Supplementary file 1 [file jcm-09-00417-s001.pdf]

Article

Circulating Arsenic is Associated with Long-Term Risk of Graft Failure in Kidney Transplant Recipients: A Prospective Cohort Study

Camilo G. Sotomayor [1,†,*], Dion Groothof [1,†], Joppe J. Vodegel [1], Tomás A. Gacitúa [1], António W. Gomes-Neto [1], Maryse C.J. Osté [1], Robert A. Pol [2], Catterina Ferreccio [3], Stefan P. Berger [1], Guillermo Chong [4], Riemer H.J.A. Slart [5], Ramón Rodrigo [6], Gerjan J. Navis [1], Daan J. Touw [7] and Stephan J.L. Bakker [1]

[1] Department of Internal Medicine, University Medical Center Groningen, University of Groningen, Groningen, The Netherlands; d.groothof@umcg.nl (D.G.); j.j.vodegel@umcg.nl (J.J.V.); t.a.gacitua.guzman@umcg.nl (T.A.G.); a.w.gomes.neto@umcg.nl (A.W.G.-N.); m.c.j.oste@umcg.nl (M.C.J.O.); s.p.berger@umcg.nl (S.P.B.); g.j.navis@umcg.nl (G.J.N.); s.j.l.bakker@umcg.nl (S.J.L.B)

[2] Division of Transplantation Surgery, University Medical Center Groningen, University of Groningen, Groningen, The Netherlands; r.pol@umcg.nl

[3] Advanced Center for Chronic Diseases, Pontifical Catholic University of Chile, Santiago, Chile; catferre@gmail.com

[4] Department of Radiology, Clínica Alemana de Santiago, Universidad del Desarrollo, Santiago, Chile; gchongm@yahoo.com

[5] Department of Nuclear and Molecular Imaging; University Medical Center Groningen, University of Groningen, Groningen, The Netherlands; r.h.j.a.slart@umcg.nl

[6] Institute of Biomedical Sciences, Faculty of Medicine, University of Chile, Santiago, Chile; rrodrigo@med.uchile.cl

[7] Department of Pharmacy and Clinical Pharmacology, University Medical Center Groningen, University of Groningen, Groningen, The Netherlands; d.j.touw@umcg.nl

† These authors contributed equally to this work

* Correspondence: c.g.sotomayor.campos@umcg.nl; Tel.: +31-50-361-0881

Table 1. Verification of linearity of the association between plasma arsenic and risk of death-censored graft failure.

	HR (95% CI)	p	BIC	$p_{comparison}$*
**Model 1a**				
Log_2 arsenic	1.31 (1.05—1.62)	0.015	914.03	
**Model 1b**				Model 1b vs model 1a: 0.69
Log_2 arsenic	1.46 (0.82—2.61)	0.20	918.14	
Log_2 $arsenic^2$	0.97 (0.82—1.14)	0.69		
**Model 1c**				Model 1c vs model 1a: 0.83
Log_2 arsenic	1.18 (0.42—3.36)	0.76	922.20	
Log_2 $arsenic^2$	1.15 (0.55—2.41)	0.71		
Log_2 $arsenic^3$	0.97 (0.85—1.11)	0.64		
**Model 2a**				
Log_2 arsenic	1.36 (1.10—1.69)	0.0053	915.82	
**Model 2b**				Model 2b vs model 2a: 0.63
Log_2 arsenic	1.56 (0.86—2.81)	0.14	919.87	
Log_2 $arsenic^2$	0.96 (0.81—1.14)	0.64		
**Model 2c**				Model 2c vs model 2a: 0.82
Log_2 arsenic	1.29 (0.45—3.76)	0.64	923.98	
Log_2 $arsenic^2$	1.12 (0.53—2.37)	0.77		
Log_2 $arsenic^3$	0.97 (0.85—1.11)	0.69		
**Model 3a**				
Log_2 arsenic	1.52 (1.20—1.92)	0.0005	916.98	
**Model 3b**				Model 3b vs model 3a: 0.48
Log_2 arsenic	1.86 (1.00—3.48)	0.050	920.76	
Log_2 $arsenic^2$	0.94 (0.79—1.12)	0.50		
**Model 3c**				Model 3c vs model 3a: 0.78
Log_2 arsenic	1.83 (0.61—5.46)	0.28	925.04	
Log_2 $arsenic^2$	0.96 (0.46—2.00)	0.90		
Log_2 $arsenic^3$	1.00 (0.88—1.14)	0.97		

*p values are for the comparison with the referent model based on a likelihood ratio test. Model 1: crude model. Model 2: adjusted for age and sex. Model 3: model 2 + fish consumption and alcohol consumption. BIC, Bayesian information criterion. HR, hazard ratio.

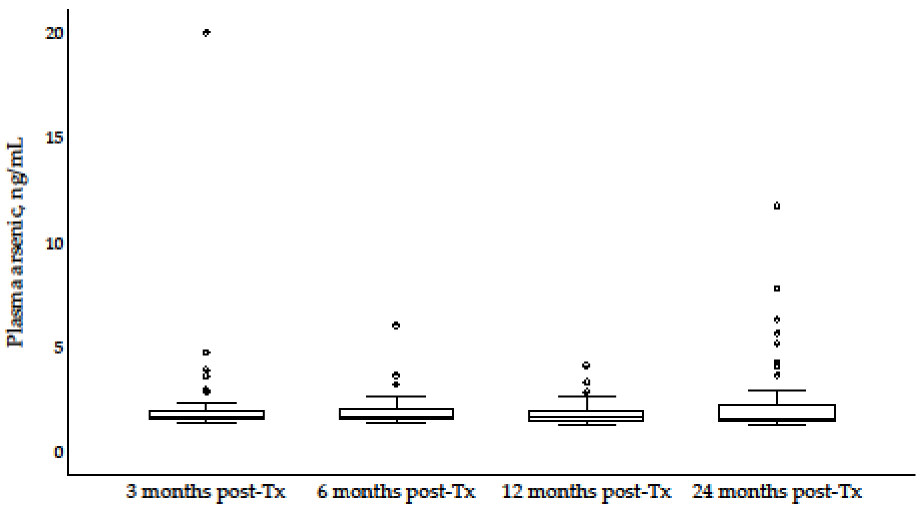

Figure 1. Plasma arsenic concentration of 46 kidney transplant recipients from the TransplantLines Prospective Cohort and Biobank Study (Eisenga, M.F.; et al. *BMJ Open* **2018,** *8,* e024502), at different follow-up visits after transplantation. Box plots show medians (interquartile range). Significance of potential change during follow-up visits was tested using the Kruskal Wallis test, which indicated no significant change over time ($p = 0.64$).
